# Is Good Clinical Practice Becoming Poor Clinical Care?

**DOI:** 10.1097/HS9.0000000000000004

**Published:** 2017-12-20

**Authors:** Steven Le Gouill, Martin Dreyling, Maria Dolores Caballero, Marc Andre, Jeannette Doorduijn, Wojciech Jurczak, Mats Jerkeman, Paolo Ghia, Pier-Luigi Zinzani, Maria Gomez Da Silva, Meletios Dimopoulos, Marek Trneny, Richard Delarue, Jan Walewski, Christian Gisselbrecht, Armando López-Guillermo, Simon Rule

**Affiliations:** 1 Centre Hospitalier Universitaire de Nantes, Nantes, France; 2 Medizinische Klinik III, Klinikum der Universität, LMU München, München, Germany; 3 Hospital Universitario de Salamanca, Salamanca, Spain; 4 CHU UCL Namur, Namur, Belgium; 5 Erasmus MC Cancer Institute, Rotterdam, The Netherlands; 6 Department of Haematology UJCM Kopernika, Kraków, Poland; 7 Lund University, Lund, Sweden; 8 Università Vita-Salute San Raffaele, Milano, Italy; 9 Bologna University, Bologna, Italy; 10 Insituto Portugues de Oncologia, Lisbon, Portugal; 11 National and Kapodistrian University of Athens, Athens, Greece; 12 Medicine University Praga, Prague, Czech republic; 13 Hopital-Necker, AP-HP, Paris, France; 14 Maria Sklodowska-Curie Institute—Oncology Center, Warszawa, Poland; 15 AP-HP, Paris, France; 16 Hospital Clínic i Provincial de Barcelona, Barcelona, Spain; 17 Plymouth University, Plymouth, UK.

Year after year, clinical research teams and investigating physicians are subjected to an exponential growth in administrative burden, paperwork, and regulation associated with clinical trials. The universal explanation for this ever-increasing workload is that it represents “good clinical practice” and is all about the safety of the patient and the integrity of the research. So whenever an investigator asks the question “Why should I fill out this document again?,” “Why should I write this sentence in the patient's record again?,” “ Why should I sign all these electronic files?,” “Why should I write the date again,” the answer is immutably identical, it is GCP.

**Figure:**
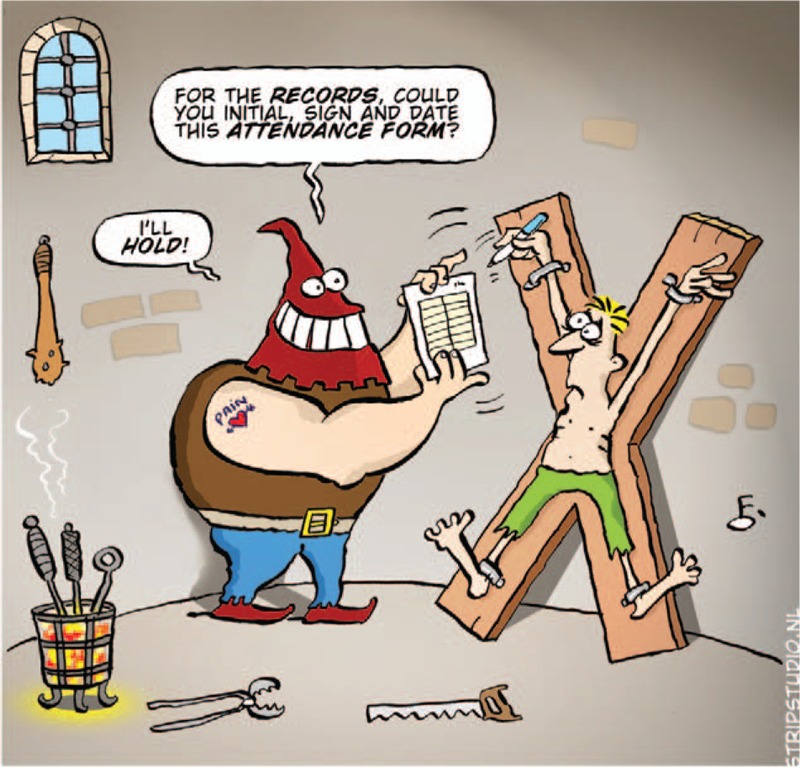


This magic sentence stops all discussion because the risk for an investigator to be seen not to be respecting “good practices” could be a death sentence for his/her center and calls into questions his/her professional integrity and that of his (or her) collaborators. Once the words GCP have been pronounced, you have to resign yourself, obey and sign (or click, or write, or listen, or ask the patient to sign here and here, there and there, in two copies if not more).

How can these nonsense, meaningless, repetitive harassments be called “clinical practice” and how on earth can they be described as being “good”? This has been a 1-way street with no actual sensible look at how this increase in bureaucracy actually benefits anyone but the industry that is inventing it. «Good clinical practice» is currently really fake GCP and should be rebaptised «fake Good clinical practice» because no one (including clinicians, promotors, clinical research organizations [CROs]) seriously believes that signing dozens and dozens of pages are ensuring quality of clinical trials and that e-mailing thousands of alert to investigators about everything (useless amendments, side effects that are not, false suspected unexpected serious adverse reactions like “progressive disease, millions of queries, etc) improves the security of patients in clinical trials. We all know that clinical trial monitoring led by a CRO research associate who knows nothing about the pathology and clinical features of the disease in question is a waste of time and that the exponential rising cost of clinical trials (and thus the rising price of new drugs) is partially due to uncontrolled growth of regulation and paperwork. Many of the people signing this letter have been involved with clinical research for years and are fortunate in having research teams around them that help with this ever-increasing workload. No one believes research today is safer, many of us believe the opposite is true as the deluge of unimportant information that follows the opening of a trial (all in the name of safety of course) means that the truly important signals are lost and dozens of pages consent forms mean that patients no longer truly understand what they are getting involved with. One unintended side effect of both uncontrolled bureaucracy and the increasing cost of clinical trials will be the rapid disappearance of independent academic clinical research and a vocation for medical research in young doctors. It is also questionable if patients will still agree to participate in clinical trials as they are overwhelmed with excessive sample collection for central labs (which does not mean quality lab) and unnecessary examinations including quality of life questionnaires to be completed at each visit which impair patients’ quality of living. Where is the good clinical practice in asking a patient to re-consent to a trial multiple times when those re-consents involve side effects seen in a drug that they never received in the randomized trial or to re-consent to having fewer blood tests.

Are European Medicines Agency, Food and Drug Administration, International Council for Harmonization, pharma companies and CROs aware of medical research evolution in real life or do they pretend to believe that everything is going well and that quality and safety is improving in clinical trials?

GCP in its present form (or its interpretations) has become inapplicable in real life and their strict application is certainly not improving either data quality or patients’ safety. This rigid administration of the current processes and the accusation that challenging them is tantamount to serious professional misconduct is setting a tone that many investigators are no longer willing to tolerate. As always, moderation is positive and excess pejorative. It is urgent for our health authorities to review the whole system in order to truly ensure patient safety and medical progress. A new approach to GCP is not only an issue in hematology and oncology; it is a concern for all those involved in clinical research. Based on the increasing burden of surreal administrational challenges, we have created an initiative in this direction and already collected the support of hundreds of colleagues from all over Europe. The list of participants will be published and accompanied by a full article describing our demands.

Help us to change real life in clinical trials by signing the “I support new GCP!” project, e-mail to your national “I support new GCP!” representative (See list below) or send an email (state your name, affiliations and write “I support new GCP”) to newgcps@gmail.com.

Prof S. Le Gouill and Pr S. Rule on behalf of the «advocacy for new GCP» board:

For Belgium and Luxemburg: Pr M. André, CHU UCL Namur, Namur , Belgium

For Czek republic and central Europe Pr M. Trneny, Medicine University Praga, Czek republic

For Denmark, Finland, Norway, and Sweden: Dr M. Jerkeman, Lund University, Sweden

For France: Dr R. Delarue, Hopital-Necker, AP-HP, Paris and Prof C. Gisselbrecht, AP-HP, France

For Germany and Austria: Prof. Dr. med. Martin Dreyling, Medizinische Klinik III, Klinikum der Universität, LMU München, Germany

For Greece: Prof Meletios A. Dimopoulos, MD, National and Kapodistrian University of Athens

For Italy: Pr P. Ghia, Università Vita-Salute San Raffaele, Milano; and Pr PL Zinzani, Bologna University

For Netherland: Dr J. Doorduijn, Erasmus MC Cancer Institute, Rotterdam, Netherland

For Poland: Prof Wojciech Jurczak, MD, PhD, Department of Haematology UJCM

Kopernika 17, 31-501 Kraków and Pr J. Walewski, Maria Sklodowska-Curie Institute—Oncology Center Warszawa

For Portugal: Dr M. Gomez Da Silva, Insituto Portugues de Oncologia, Lisbon, Portugal

For Spain: Dr Maria Dolores Caballero, Hospital Universitario de Salamanca, Salamanca and Dr A. López-Guillermo, Hospital Clínic i Provincial de Barcelona; Spain

For UK: Pr S. Rule, Plymouth University, Pymouth, UK

Or send an email (with your name, affiliations and “I support new GCP”) to: newgcps@gmail.com

